# Recent Advances in Mouse Models of Sjögren's Syndrome

**DOI:** 10.3389/fimmu.2020.01158

**Published:** 2020-06-30

**Authors:** Yunzhen Gao, Yan Chen, Zhongjian Zhang, Xinhua Yu, Junfeng Zheng

**Affiliations:** ^1^Institute of Psychiatry and Neuroscience, Xinxiang Medical University, Xinxiang, China; ^2^Priority Area Asthma & Allergy, Research Center Borstel, Airway Research Center North (ARCN), Members of the German Center for Lung Research (DZL), Borstel, Germany

**Keywords:** Sjögren's syndrome, mouse model, exocrine glands, pathogenesis, autoimmune disease

## Abstract

Sjögren's syndrome (SS) is a complex rheumatoid disease that mainly affects exocrine glands, resulting in xerostomia (dry mouth) and xerophthalmia (dry eye). SS is characterized by autoantibodies, infiltration into exocrine glands, and ectopic expression of MHC II molecules on glandular epithelial cells. In contrast to the well-characterized clinical and immunological features, the etiology and pathogenesis of SS remain largely unknown. Animal models are powerful research tools for elucidating the pathogenesis of human diseases. To date, many mouse models of SS, including induced models, in which disease is induced in mice, and genetic models, in which mice spontaneously develop SS-like disease, have been established. These mouse models have provided new insight into the pathogenesis of SS. In this review, we aim to provide a comprehensive overview of recent advances in the field of experimental SS.

## Introduction

Sjögren's syndrome (SS) is a chronic autoimmune disorder characterized by oral and ocular dryness as a result of dysfunction (1). The disease was named after Henrik Sjögren, who was the first person to identify the link among xerostomia, keratoconjunctivitis sicca, and polyarthritis (2). SS is defined as primary SS (pSS) when it presents alone and as secondary SS (2ndSS) when it presents in association with other autoimmune diseases, such as systemic lupus erythematosus (SLE) and rheumatoid arthritis (RA) (3). Similar to many other autoimmune disorders, SS is more common in females than males, with a female:male ratio of ~9:1 (4). SS predominantly affects the exocrine glands, leading to dryness in the eyes, mouth, and other organs, including the larynx, trachea, skin, and vagina (5). In addition to the involvement of exocrine glands, extraglandular manifestations, such as inflammatory arthritis, purpura, Raynaud's syndrome, interstitial lung disease, and renal disease, are observed in some patients with SS (6). Immunologically, SS is characterized by the presence of autoantibodies, such as anti-SSA/Ro and anti-SSB/La, focal lymphocytic infiltrates in the exocrine glands, production of inflammatory cytokines, and ectopic expression of MHC II molecules on glandular epithelial cells (4, 7, 8). In the past two decades, considerable progress has been achieved in our understanding of the pathogenesis of the disease (9). On the one hand, epidemiological, genetic, immunohistochemical, and *in vitro* studies with samples from SS patients and controls have shed light on the disease pathogenesis of SS (10–12). On the other hand, animal models, particularly mice, provide us with a powerful tool to elucidate the development of human SS (13).

Animal models are invaluable tools for helping us to investigate human autoimmune diseases (14). According to the strategy of disease induction, animal models can be divided into two categories: induced models, in which disease is artificially induced in animals (15), and genetic models, in which animals develop disease symptoms spontaneously due to genetic mutations or modifications (16). To date, ~20 mouse models have been established for SS, including both induced and genetic models. Although these mouse models only partially display the immunological and clinical features of SS, they are highly important for our understanding of the disease. With these mouse models, many essential factors involved in the pathogenesis of SS, including virus infection, autoreactive T cells, B cell hyper-reactivity, autoantibodies, apoptosis of glandular epithelial cells, and dysregulated homeostasis of exocrine glands, have been identified (13, 17).

In this review, we aim to provide a comprehensive overview of the recent progress in the mouse models for SS. First, we describe mouse models established for SS (Table 1). Then, we discuss recent findings in the pathogenesis of SS from these mouse models.

**Table 1:** Summary of mouse models of SS.

	**Models**	**Autoantibodies**				**Infiltration**			**Secretion Impairment**		**Ectopic expression of MHC II**	**Apoptosis of glandular epithelial cells**	**References**
		**SSA**	**SSB**	**M3R**	**120 kD α-fordin**				**Salivary**	**Tears**			
Genetic models	NOD Jcl/ICR	Yes	Yes	Yes	Yes	Yes	Yes	Pancreas	Yes	Yes	ND	Yes	(18, 19)
	NOD.B10-H2b	Yes	Yes	Yes	ND	Yes	Yes	ND	Yes	Yes	ND	ND	(20)
	C57BL/6.NOD-Aec1Aec2	Yes	Yes	Yes	ND	Yes	Yes	ND	Yes	Yes	ND	Yes	(21)
	NFS/sld	ND	ND	ND	Yes	Yes	Yes	ND	Yes	ND	Yes	Yes	(22–24)
	IQI/Jic	ND	ND	ND	Yes	Yes	Yes	Pancreas, kidneys, lungs	ND	ND	Yes	ND	(25)
	Aly/Aly mice	ND	ND	ND	ND	Yes	Yes	Liver, pancreas, lungs	ND	ND	ND	ND	(26)
	Ar KO mice	ND	ND	ND	Yes	Yes	ND	Kidney	ND	ND	ND	Yes	(27)
	RbAp48 transgenic mice	Yes	Yes	ND	Yes	Yes	Yes	ND	Yes	Yes	Yes	Yes	(28)
	Id3 KO mice	ND	ND	ND	ND	Yes	Yes	ND	Yes	Yes	ND	ND	(29)
	PI3K KO mice	Yes	Yes	ND	ND	Yes	ND	Lungs, liver, intestines	ND	ND	ND	ND	(30)
	TSP-1 KO Mice	Yes	Yes	ND	ND	ND	Yes	ND	ND	Yes	ND	Yes	(31)
	Act1 KO mice	Yes	Yes	ND	ND	Yes	Yes	Kidneys	ND	ND	ND	ND	(32)
	BAFF transgenic mice	N	N	ND	ND	Yes	Yes	Kidneys	Y	ND	ND	ND	(33)
	HTLV-1 tax transgenic mice	ND	ND	ND	ND	Yes	Yes	ND	ND	ND	ND	ND	(34)
Induced models	Ro60_480-494 induced model Ro60_274-290 induced model	Yes	Yes	ND	ND	Yes	ND	ND	Yes	N	ND	ND	(35)
	Ro60_316-335 induced model	Yes	ND	ND	ND	Yes	Yes	ND	ND	Yes	Yes	No	(36, 37)
	M3R induced model	ND	ND	Yes	ND	Yes	ND	ND	Yes	ND	ND	Yes	(38)
	MCMV induced model	ND	ND	ND	ND	Yes	Yes	ND	Yes	Yes	ND	ND	(39)
	CA II induced model	ND	ND	ND	ND	Yes	Yes	Pancreas, kidney	ND	ND	ND	ND	(40)
	SG protein induced model	ND	ND	Yes	ND	Yes	N	N	Yes	N	ND	ND	(41)

*ND, not determined; Yes, presence of the phenotype; No, absence of the phenotype*.

## Mouse Models of SS

### Genetic Models

#### Nonobese Diabetic (NOD)-Derived Strains

The nonobese diabetic (NOD) inbred strain was developed as a spontaneous mouse model of type I diabetes (T1D) by Makino et al. at Shionogi Research Laboratories in Aburahi, Japan (42). At 30–40 weeks of age, 90–100% of female and 40–60% of male NOD mice develop T1D (https://www.jax.org/strain/001976). In addition, NOD mice are characterized by inflammatory cell infiltration in the exocrine glands and impaired secretion of saliva and tears, which resemble the symptoms of SS (43, 44). Furthermore, many SS-associated autoantibodies, such as anti-SSA/Ro, anti-SSB/La, anti-120 kD α-fodrin, and anti-muscarinic type 3 acetylcholine receptors (M3Rs), are present in the serum of NOD mice (44–46). Because NOD mice develop both autoimmune diabetes and SS-like disease, they represent a mouse model of 2ndSS.

To establish a mouse model of pSS, researchers generated NOD.B10. *H*2^b^, a congenic mouse strain, by replacing the major histocompatibility complex (MHC) I-*A*^*g*7^ molecule of NOD mice with MHC I-A^b^ from the B10 strain (47, 48). Because I-*A*^*g*7^ is essential for the development of diabetes and insulitis in NOD mice, NOD.B10. *H*2^b^ mice are protected from autoimmune diabetes but still develop SS-like symptoms, making these mice an ideal model of pSS (49). In addition to NOD.B10. *H*2^b^, another NOD-derived strain used as a mouse model of pSS is C57BL/6.NOD-*Aec1Aec2*, which has a C57BL/6 background but carries two autoimmune exocrinopathy loci from NOD mice (46, 50). C57BL/6.NOD-*Aec1Aec2* mice develop many clinical and immunological features resembling pSS, such as impaired production of saliva and tears, increased salivary protein content, aberrant proteolytic enzyme activity, glandular lymphocytic focal infiltrates, and the appearance of autoantibodies (46, 50).

As mouse models of SS, NOD-derived strains have been extensively investigated, and many factors, including T cells, B cells, various cytokines, and dysregulated homeostasis in exocrine glands, have been shown to contribute to the development of the disease (51–56). Based on the findings from those studies, a three-phase hypothesis of disease manifestation in NOD-derived strains has been proposed. In the first phase (0–8 weeks of age), several aberrant genetic, physiological, and biochemical activities occur prior to the initiation of disease. The second phase (8–16 weeks of age) is characterized by inflammatory cell infiltration in exocrine glands and the production of autoantibodies and pro-inflammatory cytokines. Finally, in the third phase (>16 weeks of age), the function of the salivary and lacrimal glands (LG) is impaired due to autoimmune-mediated attack (18, 19, 57). The unique advantage of NOD and NOD-derived models is that disease symptoms develop spontaneously as a polygenic trait, which is similar to the development of human SS. However, due to the high heterogeneity of SS in both symptoms and pathogenesis, NOD and NOD-derived mouse models might resemble the disease in a subgroup of human patients.

#### NFS/sld Mice

NFS/*sld* mice bear a mutation in an autosomal recessive gene (sublingual gland differentiation arrest, *sld*), which inhibits the acinar cells of the sublingual gland from differentiating into mucus-secreting cells (21). In 2013, the *sld* mutation was identified as two intronic CA repeats within the mucin 19 (MUC19) gene; this mutation promotes mRNA decay (20). Notably, when NFS/*sld* mice were thymectomized 3 days after birth, they spontaneously developed SS-like disease (58) and, thus, represent a mouse model of pSS. The lymphocytic infiltrates in exocrine glands are dominated by CD4^+^ T cells with fewer CD8^+^ T cells and B cells (58). In 1997, Haneji et al. identified alpha-fodrin (α-fodrin), a 120 kDa salivary gland–specific protein as a disease-relevant autoantigen in this model, suggesting a novel autoantigen in human SS (59).

Several studies have been performed to elucidate the pathogenesis of the pSS-like disease in NFS/*sld* mice. For example, Ishimaru et al. reported that estrogen deficiency accelerates autoimmune exocrinopathy in these mice, indicating that estrogen plays a protective role in SS (60). In addition, an increase in the number of apoptotic epithelial duct cells in the salivary glands was observed in aged NFS/*sld* mice, and this increase was further enhanced by estrogen deficiency (60, 61). In addition to the increased number of apoptotic epithelial cells, aged salivary glands in the NFS/*sld* mice showed increased levels of the apoptotic cleavage product of 120 kD α-fodrin and autoimmunity to the organ-specific antigen (61), suggesting a role for the apoptosis of epithelial cells in disease pathogenesis. Furthermore, a role of the co-stimulatory molecule CD86 has been suggested by the finding that intraperitoneal administration of anti-CD86 antibody inhibits the development of autoimmune lesions and the production of autoantibodies against 120 kD α-fodrin (62).

The NFS/sld model has also been applied to evaluate the role of environmental factors or therapeutics. For example, neonatal NFS/sld mice without thymectomy exposed to an apoptotic dosage of 2,3,7,8-tetrachlorodibenzo-p dioxin (TCDD), an herbicide regarded as a potential environmental trigger for SS, developed autoimmune lesions in the salivary glands and other organs, suggesting a role of TCDD in the disruption of T cell tolerance (63). In addition, with NFS/sld mice, both cyclosporine, a potent immunosuppressant (64), and cepharanthine, a biscoclaurine alkaloid, were shown to be effective in the treatment of experimental SS (65).

Because neonatal thymectomy impairs the expansion of regulatory T cells (66), this model suggests an essential role of regulatory T cells in the development of the disease. Furthermore, NFS/sld mice represent a powerful tool for investigating the pathogenesis of SS associated with autoantibodies against alpha-fodrin. However, thymectomy, an indispensable procedure, limits its application.

#### IQI/Jic Mice

Similar to NOD mice, IQI/*Jic* mice were also developed from outbred imprinting control region (ICR) mice in Japan (67). IQI/*Jic* mice are autoimmune-prone and susceptible to mercuric chloride-induced production of anti-nucleolar antibody (ANA). Moreover, female IQI/*Jic* mice show focal lymphocyte infiltration and tissue destruction in the salivary glands (SG) and LG. Similar to human SS, to which females are more susceptible than males, more than 80% of female IQIL/*Jic* mice spontaneously developed sialadenitis at 6 months of age although male mice showed only mild salivary lesions (22). In addition to exocrine gland infiltration, IQIL/*Jic* mice show lymphocytic infiltrates in the lungs, pancreas, and kidneys (22), suggesting that the relevant autoantigen(s) might not be tissue-specific. This hypothesis is supported by the identification of kallikrein 13 (Klk13), a protein expressed in multiple tissues as an autoantigen in IQIL/*Jic* mice (68). In addition to clinical symptoms, immunological features associated with human SS, including antinuclear autoantibodies and aberrantly expressed MHC II molecules on glandular epithelial cells, are present in IQIL/*Jic* mice (22).

When IQI/*Jic* mice were thymectomized (Tx) 3 days after birth to eliminate CD4^+^CD25^+^ regulatory T (Treg) cells, they developed more severe autoimmune lesions in the lacrimal glands. In addition, the transfer of CD25^+^ splenic T cells from normal mice to Tx IQI/*Jic* mice could inhibit the development of the disease (23, 69), suggesting an inhibitory role of Treg cells in this model. In addition, a role of dendritic cells (DCs) has been suggested in these mice because MHC II^+^CD11c^+^B7-2 (CD86^+^) DCs were detected in the salivary and lacrimal glands of the mice at 4 weeks of age, and these DCs clustered to form network-like structures at lesion regions (24).

Like NOD mice, IQI/Jic mice develop SS-like disease as a polygenic trait spontaneously resembling the development of human SS. However, the age of onset in this mouse model is at least 9 months, which limits its application. In addition, non-exocrine organs, such as the pancreas, kidneys, and lungs, are also affected in IQI/Jic mice, suggesting that this is a mouse model for 2ndSS.

#### Aly/aly Mice

*A*ly/*aly* mice are homozygous for an autosomal recessive alymphoplasia (aly) mutation within the NF-kappa B-inducing kinase (NIK) gene, which leads to the lack of both lymph nodes and Peyer's patches as well as to disorganization of the thymus and spleen (70, 71). Although mature T and B cells are present, aly/aly mice are deficient in both cellular and humoral immune responses (70). Interestingly, *aly/aly* mice exhibited chronic inflammatory cell infiltration in multiple organs, including the salivary and lacrimal glands, pancreas, and lungs (72). The inflammatory infiltrates in exocrine glands are predominantly composed of CD4^+^ T cells, and the transfer of T cells from the spleen of *aly/aly* mice to *Rag2*^−/−^ mice induced inflammation in the exocrine glands and pancreas (72), suggesting an essential role for autoreactive T cells in this model. However, there are no detectable autoantibodies against nuclear components or salivary gland proteins in the serum of aly/aly mice (72). Notably, aly/aly mice develop systemic disease that can involve multiple organs, and they are also used as animal models for pancreatitis (73), dermatitis (74), and osteoporosis (75).

#### Ar KO Mice

SS is highly prevalent in postmenopausal women, implying that estrogen deficiency contributes to the development of this disease. To explore the role of estrogen deficiency in SS, Shim et al. investigated mice deficient in aromatase cytochrome P450, an enzyme catalyzing the formation of estrogens from C19 steroids (25, 76, 77). Aromatase enzyme knockout (*Ar* KO) mice show mild splenomegaly and lymphadenopathy as a result of hypercellularity in the bone marrow with the overproduction of mature granulocytes and B cells (77). Moreover, *Ar* KO mice develop spontaneous autoimmune manifestations, such as proteinuria and severe leukocyte infiltration in the exocrine glands and kidneys, which partially resemble SS (77, 78). Additionally, as observed in human SS, *Ar* KO mice produce proteolytic fragments of 120 kD α-fodrin in the salivary glands, and their serum contains autoantibodies against 120 kD α-fodrin (77). Taken together, these observations indicate that *Ar* KO mice are an ideal mouse model for investigating the role of estrogens in the pathogenesis of SS. One limitation of this mouse model could be the age of onset, where *Ar* KO mice develop SS-like disease at the age of more than 12 months.

#### RbAp48 Transgenic Mice

Retinoblastoma-associated protein 48 (RbAp48) is a multifunctional protein that binds to transcription factors and kinases to control cell growth and apoptosis (79). In mice, ovariectomy induces tissue-specific apoptosis in exocrine glands, and RbAp48 expression is increased in apoptotic glandular epithelial cells (80). Moreover, overexpression of RbAp48 induced apoptosis in epithelial cells with p53 phosphorylation (on Ser9) and α-fodrin cleavage, and knockdown of RbAp48 by siRNA inhibited p53-mediated apoptosis (80), suggesting that RbAp48 is functionally involved in the estrogen deficiency–mediated exocrine gland–specific apoptosis. To further explore the role of RbAp48 in the pathogenesis of SS, Ishimaru et al. generated and investigated *RbAp48* transgenic mice (*RbAp48-tg*) overexpressing the gene in a salivary gland–specific manner (80, 81). As expected, the *RbAp48-tg* mice showed apoptosis in the exocrine glands but not in other organs (80). Moreover, the *RbAp48*-*tg* mice developed autoimmune exocrinopathy resembling Sjögren's syndrome, including lymphocytic infiltration in the exocrine glands; the impairment of saliva and tear secretion; the ectopic expression of MHC II molecules on glandular epithelial cells; and the production of autoantibodies against SSA/Ro, SSB/La, and 120 kD α-fodrin (81). In addition, autoimmune lesions in the exocrine glands can be induced by the transfer of lymph node T cells from *RbAp48*-Tg mice into *Rag2*^−/−^ mice, demonstrating a pathogenic role of autoreactive T cells in this model (81). RbAp48 transgenic mice represent an ideal model for investigating the role of apoptotic glandular epithelial cells in SS. However, this model is fully dependent on the artificial overexpression of *RbAp48*, a scenario that does not exist in human patients.

#### Id3 KO Mice

Id3, a member of the basic-helix-loop-helix (bHLH) transcription factor family, is involved in T and B cell selection during lymphocyte development (82, 83). Id3-deficient (*Id3* KO*)* mice show reduced B cell reactivity and a low percentage of single-positive cells in the thymus (83, 84). In 2004, Li et al. reported *Id3* KO mice as a novel mouse model of pSS (85). At the age of 2 months, the exocrine glands of the *Id3* KO mice show severe lymphocyte infiltration, which is mainly composed of CD4^+^ T cells followed by CD8^+^ T cells and B220^+^ B cells (85). Subsequently, the secretion of saliva and tears is significantly decreased in *Id3* KO mice, resulting in dryness in the mouth and eyes. At the age of 1 year, the *Id3* KO mice show anti-SSA/Ro and anti-SSB/La antibodies in the serum.

Both T and B cells play essential roles in the development of pSS-like disease in Id3 KO mice. On the one hand, the elimination of T cells in *Id3* KO mice prevented lymphocyte infiltration into the exocrine glands and the impairment of saliva and tear secretion (85), suggesting an indispensable role of T cells. On the other hand, B cell ablation using an anti-CD20 monoclonal antibody ameliorated the pSS-like disease in *Id3* KO mice (26). Therefore, *Id3* KO mice represent a mouse model in which both cellular and humoral autoimmunity contribute to disease pathogenesis, which mimics the situation in human SS. Notably, pSS is not associated with genetic variants in the *Id3* gene (86), suggesting potential differences between this model and human disease.

#### T Cell–Specific PI3K KO Mice

Phosphoinositide 3-kinase (PI3K), a member of the lipid kinase family involved in diverse cell functions, catalyzes the production of 3-phosphorylated phosphoinositides and serves as a second messenger downstream of many cellular receptors (87). Class IA PI3K, which consists of a catalytic subunit with a molecular weight of 110 kD and a regulatory subunit, plays an important role in the function of lymphocytes (88). To investigate the role of Class IA PI3K in T cells, Oak et al. generated a T cell–specific class IA *PI3K*-deficient mouse strain (89). Although class IA *PI3K*-deficient T cells show only partial defects in function, the T cell–specific class IA *PI3K*-deficient mice develop a pSS-like disease. These mice develop corneal opacity and eye lesions resulting from irritation and excessive scratching at an age of 4–12 months (89). In addition, antinuclear, anti-SSA/Ro, and anti-SSB/La antibodies are detectable in the serum of 1 year-old T cell–specific class IA *PI3K*-deficient mice (89). Histologically, the lacrimal glands of these mice are characterized by destruction of the acinar structure, ductal hypertrophy, and infiltration by lymphocytes, which are composed predominantly of CD4^+^ T cells. In contrast to the lacrimal glands, the salivary glands of T cell–specific class IA *PI3K*-deficient mice show levels of inflammatory cell infiltration similar to those in the corresponding controls. Taken together, these findings suggest that T cell–specific class IA *PI3K*-deficient mice represent a mouse model for autoimmunity-mediated xerophthalmia (89).

#### TSP-1 KO Mice

Thrombospondin-1 (TSP-1) is a 450 kD matricellular protein that activates latent transforming growth factor (TGF)-β *in vivo* and *in vitro* (27, 90). Consistent with this role, TSP-1-deficient (*TSP-1* KO) mice show similar but less severe pathological abnormalities than TGF-β 1-null mice (91). By the age of 24 weeks, *TSP-1* KO mice develop SS-like disease characterized by increased epithelial cell apoptosis, inflammatory infiltrates in the lacrimal glands, SSA and SSB autoantibodies, and impaired secretion of tears (92). Notably, the development of SS-like disease in *TSP-1* KO mice was suppressed by topical application of a TSP-1-derived peptide (KRFK) (93). *In vitro* findings have demonstrated that the KRFK peptide activates TGF-β and, thus, reduces the expression of co-stimulatory molecules on DCs, driving DCs toward a tolerogenic phenotype and ultimately increasing the proportion of Treg cells (93). A limitation of this model is that it only resembles the xerophthalmia of SS patients.

#### Act1 KO Mice

Act1 plays an important role in the homeostasis of B cells by negatively regulating CD40- and BAFF-mediated signaling (28, 94). As expected, *Act1*-deficient (*Act1* KO) mice show B cell hyper-reactivity and develop systemic autoimmune disease resembling human SS in association with SLE-like nephritis (95). By the age of 6 months, *Act1* KO mice develop oral inflammation with enlarged submaxillary glands and lymph nodes localized proximal to those glands. Furthermore, these mice show severe lymphocyte infiltration in the lacrimal and salivary glands, anti-SSA/Ro and anti-SSB/La antibodies in the serum, and impaired secretion of saliva and tears (95). In addition, these mice develop glomerulonephritis (95), making them a useful model for 2ndSS associated with SLE.

With regard to the pathomechanism of SS-like disease in *Act1* KO mice, Qian et al. demonstrated that Act1 modulates the survival of autoreactive B cells by regulating BAFF-mediated cell survival and affecting autoantibody production by modulating the CD40-mediated T cell–dependent humoral response (95). In addition, Johnson et al. demonstrated that T cells are necessary for the development of systemic autoimmune disease in *Act1* KO mice (96).

#### ERdj5 KO Mice

The endoplasmic reticulum (ER) serves as the protein-processing factory responsible for proper protein processing, folding, and trafficking. The chaperone protein ERdj5, an ER-resident protein containing DnaJ and thioredoxin domains, is required for the translocation of misfolded proteins during ER-associated protein degradation (29, 97), the regulation of calcium ion homeostasis in the ER (98), the correct folding of the LDL receptor (99), and the sensitization of neuroblastoma cells to ER stress-induced apoptosis (100). Mice deficient in ERdj5 showed an activated ER stress response in salivary glands (101), suggesting that ERdj5 contributes to ER protein quality control.

It has been shown that ERdj5 is highly expressed in the minor SGs of SS patients, particularly in ductal and acinar epithelium and in the infiltrating mononuclear cells (30). Furthermore, the levels of ERdj5 correlate with the severity of inflammation and anti-SSA/Ro positivity (30), suggesting that ERdj5 might be involved in the development of SS. Notably, ERdj5 KO mice with a129/Sv genetic background spontaneously developed many SS-like features, including inflammation in SGs, increased inflammatory cytokines, apoptosis in SGs, impaired saliva secretion, and production of anti-SSA/Ro and anti-SSB/La autoantibodies (30). Consistent with that in human SS, disease manifestation in ERdj5 KO mice is associated with sex, where female mice show more prevalent and severe disease than age-matched males. The inflammatory infiltrates in SGs of the ERdj5 KO mice mainly include B cells and T cells with an approximate B/T ratio of 2:1 that remains unaltered with aging. The ERdj5 KO mice have many immunological, histological, and clinical features of human SSSS, but this model only affects SGs and not LG.

#### BAFF-tg Mice

B cell activating factor (BAFF) is a member of the TNF superfamily that acts as a powerful regulator of B cell survival and proliferation (102). Serum levels of BAFF are increased in SLE, SS, and the corresponding animal models, and treatment of lupus-prone mice with the BAFF decoy receptor prevents the onset of disease (103), suggesting an essential role of BAFF in systemic autoimmune diseases. *BAFF* transgenic C57BL/6 (*BAFF*-*tg*) mice overexpressing BAFF develop systemic autoimmune symptoms characterized by B cell hyper-proliferation, high levels of rheumatoid factor and anti-DNA antibody production, SLE-like nephritis, lymphocyte infiltration in the lacrimal glands, sialadenitis, and impaired salivary secretion (104). Unlike the *Act1* KO mice, the *BAFF*-*tg* mice show no disease pathology in the lacrimal glands and do not produce anti-SSA/Ro and anti-SSB/La antibodies (31, 104). Notably, *BAFF*-*tg* mice lacking LT-beta and, thus, lacking MZ B cells do develop nephritis but not sialadenitis (105), suggesting that MZ B cells contribute to the dysfunction of salivary glands in the *BAFF*-*tg* mouse model of SS. Similar to *Act1* KO mice, *BAFF*-*tg* mice represent a mouse model for SS associated with SLE.

#### HTLV-1 Tax Transgenic Mice

Circumstantial evidence suggests that the etiology and pathogenesis of SS are associated with retroviruses, including human T cell lymphotropic virus 1 (HTLV-1) (106). In 1987, Hinrichs et al. generated transgenic mice expressing the *HTLV-1 tax* gene under the control of viral long terminal repeat (LTR) sequences (107). In addition to developing neurofibromas, the *HTLV-1 tax* transgenic mice developed SS-like pathology (32, 107). Several weeks after birth, the *HTLV-1 tax* transgenic mice showed ductal epithelial cell proliferation in the exocrine glands without functional impairment and exhibited lymphocyte infiltration. By 8 weeks of age, the *HTLV-1 tax* transgenic mice showed abnormal ductal cell proliferation as well as severe lymphocytic infiltration into the exocrine glands (32). Thus, the *HTLV-1 tax* transgenic mice represent a mouse model for investigating the mechanism underlying the association between HTLV-1 and SS.

#### STAT3 KO and IκB-ζ KO Mice

IκB-ζ is a member of the nuclear IκB family of proteins that regulates gene expression via association with NF-κB (108). Interestingly, IκB-ζ KO mice spontaneously develop lymphocytic inflammation with mainly CD4^+^ T cells in the lacrimal glands, conjunctiva, and facial skin by the age of 8 weeks regardless of sex (109). With the progression of inflammation, high titers of anti-SSA and anti-SSB autoantibodies as well as impaired ear secretion could be observed in the IκB-ζ KO mice. In addition, aged IκB-ζ KO mice often exhibited interstitial pneumonia, a frequent complication of SS (109). Notably, in the IκB-ζ KO mice, epithelial cell apoptosis preceded lymphocyte infiltration, supporting an etiological role of epithelial cell apoptosis in human SS.

Signal transducers and activators of transcription 3 (STAT3) are regulators of IκB-ζ expression (110) and are required for the expression of IκB-ζ in the epithelial cells of lacrimal glands (109). Interestingly, both epithelial cell–specific IκB-ζ KO mice and epithelial cell–specific STAT3 KO mice show SS-like phenotypes, such as lymphocyte-infiltrating periocular dermatitis, dacryoadenitis, conjunctivitis, autoantibodies against SSA and SSB, and impaired tear secretion, suggesting a role of dysregulated homeostasis of epithelial cells in the pathogenesis of the disease (109). However, a limitation of STAT3 KO and IκB-ζ KO mice is that only lacrimal glands are affected.

### Induced Mouse Models

#### Salivary Gland Protein–Induced Mouse Model

Similar to induced models of many other autoimmune diseases (14), the first induced model of SS was established by immunizing animals with tissue/cell extracts. In 1974, White et al. reported an experimental rat model of sialadenitis induced by immunization with allogeneic submandibular gland homogenate emulsified in Freund's complete adjuvant (FCA) (111). By immunizing mice with syngeneic submandibular gland homogenate, Yasunori et al. induced the first experimental model of autoallergic sialadenitis resembling human SS (112). By 4 weeks after immunization, the SL/Ni mice showed “marked lymphoid cell infiltration in the submandibular glands with high incidence and proliferation of duct epithelial” (112). In addition to SL/Ni mice, C57BL/6 mice are susceptible to the development of salivary gland protein-induced SS-like disease (113, 114). After immunization, C57BL/6 mice show enhanced apoptosis and increased expression of M3R in the salivary glands. Furthermore, immunized C57BL/6 mice produce autoantibodies against M3R and show inflammatory infiltration in the salivary glands and reduced saliva secretion (114, 115). In addition, an enhanced Th17 cell response has been observed in the cervical lymph nodes and salivary glands of C57BL/6 mice during the development of disease (114). In contrast, IL-17-deficient mice are completely resistant to the development of disease (114), suggesting an indispensable role of IL-17 in this model. Because salivary gland proteins represent a complex mixture of antigens, a limitation of this model is the unknown pathogenic autoantigen(s).

#### Carbonic Anhydrase II-Induced Mouse Model

Carbonic anhydrase II (CAII) is a human α carbonic anhydrase that catalyzes the reversible hydration of carbon dioxide (116). Although autoantibodies against CAII are a hallmark of autoimmune pancreatitis (117), they are also associated with other autoimmune diseases, including SS and SLE (118, 119). Interestingly, PL/J (*H-2*^*u*^) mice immunized with CAII showed severe lymphocyte infiltration around the intercalated and intralobular ducts in the salivary and lacrimal glands (120). In addition, similar lymphocyte infiltration was observed in the kidneys and pancreas of a small number of immunized mice (120). Consistent with the observation that human SS is associated with HLA (33, 121), the development of the CAII-induced mouse model is associated with the *H-2*^*s*^ and *H-2*^*u*^ haplotypes. Although PL/J (*H-2*^*u*^) mice immunized with CAII represent a novel induced mouse model for SS, its relevance to human disease might be low because CAII is unlikely to be a major target antigen in patients (122).

#### M3R Immunization

M3R is one of five members of the family of muscarinic receptors that mediate many physiological responses, such as smooth muscle contraction, heart rate, and glandular secretion (123). Because M3R is involved in the regulation of the secretory function of exocrine glands, it is conceivable that autoantibodies against M3R with an antagonistic effect can impair the secretion of saliva and tears (34, 124, 125), making M3R a putative autoantigen in SS. To determine the pathogenicity of autoimmunity against M3R *in vivo*, Iizuka et al. immunized M3R-deficient (*M3R*^−/−^) mice with a six-valent (N-terminus 1, N-terminus 2, N-terminus 3, 1st extracellular loop, 2nd extracellular loop, and 3rd extracellular loop) mixture of murine M3R peptides and then transferred splenocytes from the immunized mice into *RAG1*^−/−^ mice (126). As expected, the recipient *RAG1*^−/−^ mice produced high levels of anti-M3R antibodies, suggesting strong immune responses against M3R. Furthermore, the recipient mice showed apoptotic glandular epithelial cells and severe lymphocyte infiltration with predominantly CD4^+^ T cells in the salivary glands as well as impaired secretion of saliva, resembling human SS. Therefore, this M3R-induced mouse model supports the hypothesis that M3R is a putative autoantigen in human SS. Notably, the transfer of CD3^+^ T cells isolated from immunized *M3R*^−/−^ mice into *Rag1*^−/−^ mice was sufficient to induce SS-like disease, suggesting that autoreactive T cells against M3R are the key player in the pathogenesis of this model (126). This hypothesis is further supported by another study in which Tahara et al. showed that administration of A213, a potent and selective *ROR*γ*t* antagonist that inhibits the differentiation of CD4^+^ T cells into Th17 cells, could prevent M3R-induced SS-like disease (127). Taken together, these findings indicate that, in this mouse model, T cell responses—particularly Th17 responses—to M3R are pathogenic, but autoantibodies against M3R peptides do not play an essential role in the development of disease. The non-pathogenic role of autoantibodies against the M3R peptide was also shown in another study, in which Chen et al. immunized BALB/c mice with a peptide of the 2nd extracellular loop of murine M3R that contains the binding site of the ligand. Although the immunized mice generated autoantibodies against the peptide of the 2nd extracellular loop of murine M3R, they showed no histological or pathological abnormalities in the exocrine glands (127).

#### Ro60 Peptide-Induced Mouse Model

Anti-SSA/Ro autoantibodies, a diagnostic biomarker for SS, are present in the serum of ~75% of patients with SS (128). In 2005, Scofield et al. immunized BALB/c mice with Ro60 peptides, which contain epitopes recognized by SSA autoantibodies from SS patients (41). After repetitive immunization with the Ro60_480-494 or Ro60_274-290 peptide emulsified in CFA, mice developed both anti-SSA/Ro and anti-SSB/La antibodies, suggesting intermolecular epitope spreading. By 263 days after the first immunization, the mice showed lymphocytic infiltrates composed of both T cells and B cells in the salivary glands and a significantly decreased salivary flow rate (41). Unlike the BALB/c strain, the DBA-2, PL/J, SJL/J, and C57BL/6 strains are resistant to Ro60_274-290 peptide-induced SS-like disease; the SJL/J strain showed no immune responses to the peptide, and the other three strains reacted to the peptide but showed no disease symptoms (129).

In 2017, Zheng et al. reported another mouse model of SS induced by immunizing mice with the Ro60 peptide (130) in which mice are immunized with the Ro60_316-335 peptide containing a dominant T cell epitope (131). A single immunization with the Ro60_316-335 peptide emulsified with TiterMax as an adjuvant induced the production of autoantibodies recognizing multiple lacrimal proteins, suggesting intermolecular epitope spreading. By 12 weeks after immunization, C3H/He mice showed lymphocyte infiltration into the lacrimal glands and impaired tear secretion, but no histological or pathological abnormalities were observed in the salivary gland. Among the four mouse strains, the C3H/He and BALB/c strains were susceptible but the DBA/1J and C57BL/6J strains were resistant to the Ro60_316-335 peptide–induced SS-like disease (131). Notably, ectopic expression of MHC II molecules on the glandular epithelial surface, a feature of human SS, has been observed in this model as a pre-symptomatic event, and the ectopic expression of MHC II molecules is mediated by the adjuvant (132). In addition, depletion of B cells with an anti-CD20 monoclonal antibody could prevent mice from developing SS-like disease, suggesting an essential role of B cells in disease pathogenesis (130). Given that anti-Ro60 autoantibodies are not pathogenic, the pathogenic mechanism of the Ro60 peptide-induced models remains unclear.

#### MCMV-Induced Mouse Model

Human CMV virus replicates mainly within the ductal epithelium of the salivary glands although murine CMV (MCMV) virus replicates primarily within the acinar epithelial cells of the submandibular glands (40, 133). To investigate the relationship between CMV and human SS, Fleck et al. induced MCMV infection by intra-peritoneal injection into four mouse strains: C57BL/6, *Fas*-deficient B6-lpr/lpr, *TNFRI-*deficient B6-tnfr1^0/0^, and B6-tnfr1^0/0^-lpr/lpr (134). Mice of all of these strains developed acute sialadenitis at 28 days after infection, but only the B6-lpr/lpr mice showed SS-like symptoms, such as severe salivary gland inflammation and anti-SSA/Ro and anti-SSB/La antibody production at 100 days after infection (135). Because the B6-lpr/lpr strain has an autoimmune background, this finding suggests that the MCMV-induced SS-like disease is a result of the interaction between viral infection and preexisting autoimmunity. This hypothesis is further supported by another study in which SS-like disease was induced by MCMV infection in another autoimmune-prone strain, NZM2328 (136). This virus-induced SS-like disease in mice represents a mouse model for 2ndSSc, and it is useful to explore the role of viral infection in the etiology and pathogenesis of SS. However, this model can only be induced in autoimmune-prone strains, which limits its utility.

#### Adenovirus 5 (AdV5)-Induced Mouse Model

Salivary glands in 30–40% of patients with SS develop ectopic lymphoid structures (ELS) that contain T and B lymphocytes, follicular dendritic cells, and auto-reactive plasma cells (137, 138). To explore the key molecular and cellular events regulating the formation of ELS, Bombardieri et al. induced replication-deficient adenovirus-5 (Adv5) infection in the submandibular glands of C57BL/6 mice by retrograde excretory duct cannulation (139). Adv5 delivery in the submandibular glands (SMGs) could induce the formation of ELS and many other SS-like features in a dose-dependent manner. By week 3 post-cannulation, the mice strongly showed several cardinal features of SS, including infiltration of T and B cells, formation of ELS, development of anti-nuclear antibody (up to 75% of mice), and reduction in salivary flow (139). Immunofluorescence staining showed that Adv5-induced ELS in C57BL/6 cells is characterized by T/B cell segregation, differentiation of high endothelial venules, development of follicular dendritic cell networks and formation of GL7+ germinal centers. Further studies suggested that Adv5-induced CXCL13 and CCL19 are two important chemokines for the formation of ELS (38, 140). In addition, Adv5 infection could induce the formation of a network composition by a population of podoplanin-positive (pdpn+) stromal cells that preceded lymphocyte infiltration in the tissue mediated by paracrine and autocrine signals, which were mainly regulated by IL13 (141). Once lymphocytes were recruited, the production of the tissue local cytokines IL22 and lymphotoxin expanded and stabilized the initial fibroblast network. Depleting the immunofibroblasts or inhibiting the pdpn+ network regulatory events resulted in abrogation of local pathology (141). The Adv5 delivery–induced model provides a unique tool to explore the mechanisms underlying ELS formation and the pathology of ELS-associated SS. For example, using this model, Nayar et al. showed that the Pi3Kδ pathway within the glands contributes to ELS formation and disease manifestation (35).

## What Have We Learned From These MOUSE Models of SS

**Figure 1:**
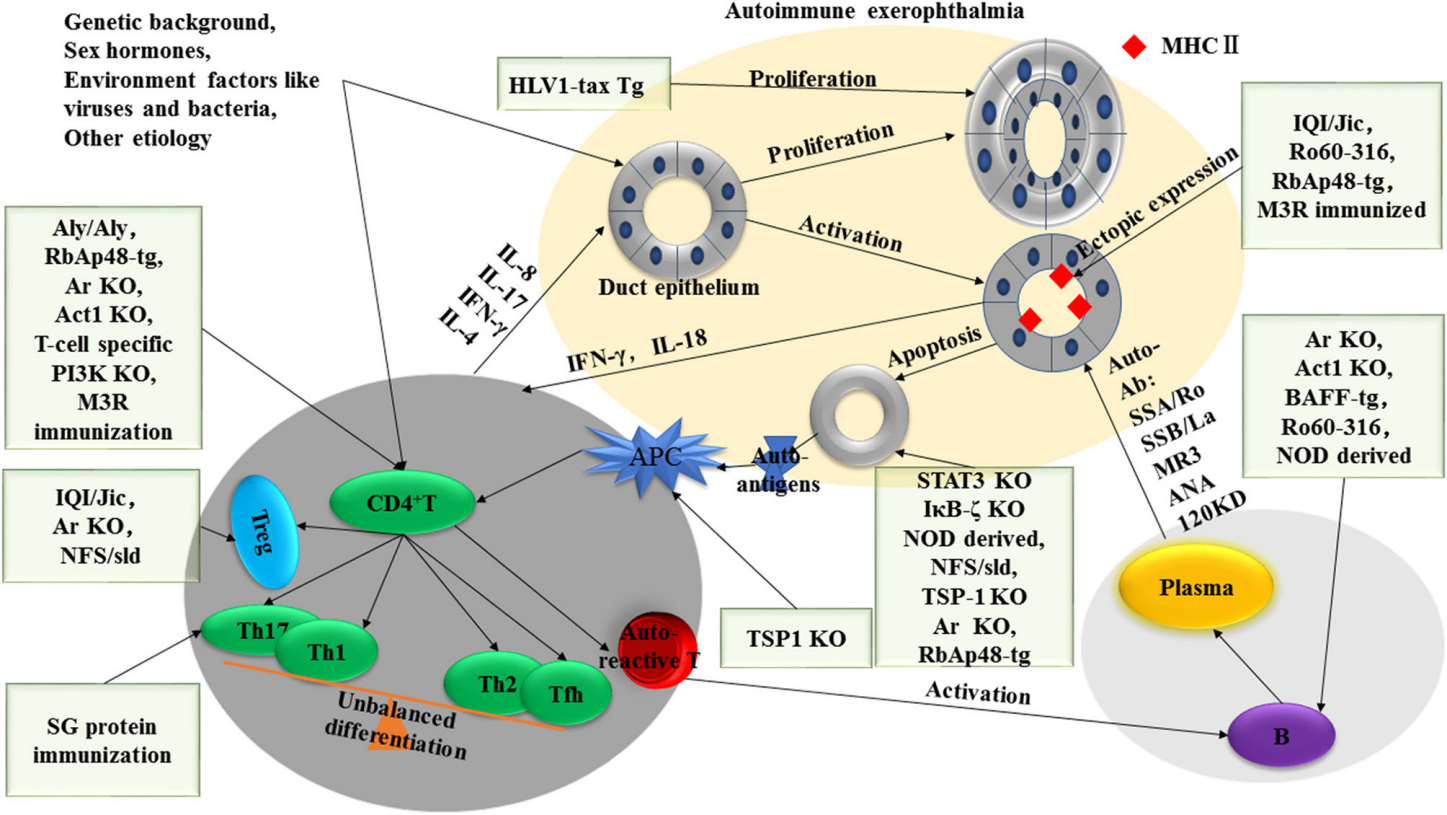
Dysregulated homeostasis in both immune cells and exocrine glands is involved in the development of SS-like disease in mice. On the one hand, abnormal proliferation (HLV1-tax-Tg model), apoptosis of exocrine gland epithelial cells (STAT3 KO, IκB-ζ KO, NOD-derived mice, NFS/sld, TSP-1 KO, Ar KO, and RbAp48-tg mouse models), and ectopic expression of MHC II in the exocrine gland (IQI/Jic, Ro60-peptide immunization, RbAp48-*tg*, and M3R-immunized models) are suggested to be involved in disease pathogenesis by the corresponding mouse models. On the other hand, APCs (TSP1 KO), CD4 T cells (Aly/Aly, RbAp48-tg, Ar KO, Act1 KO, PI3K KO, and M3R immunization mouse models), B cells (Ar KO, Act1 KO, NOD, Ro60-peptide immunization, BAFF-tg), Treg cells (Ar KO, NFS/sld, IQI/Jic), and cytokines (SG protein immunization) have been suggested to play a role in the development of disease in corresponding mouse models.

The mouse models of SS have extensively elucidated the pathogenesis of the human disease. According to their roles in the mouse models of SS, factors that contribute to the development of disease can be divided into two major categories: alterations in the immune system and dysregulation of exocrine gland homeostasis (Figure 1).

### Alterations in the Immune System

#### Role of DCs

DCs, a key type of antigen-presenting cells (APCs), are essential for the initiation of adaptive immune responses, including the generation of autoreactive T cells and regulatory T cells (Tregs). The essential role of DCs in the development of SS-like disease is supported by several mouse models. For example, in mouse models induced by immunization with antigens, such as salivary gland proteins (112), the CAII protein (120), or the M3R (126) and Ro60 peptides (126, 130), it is clear that DCs are involved in processing and presenting antigens to T cells. In genetic models, a role of DCs is suggested in *TSP-1* KO mice, where *TSP-1* deficiency decreases the activation of TGF-β, thus enhancing the expression of co-stimulatory molecules on DCs, leading to autoimmunity and autoimmune diseases (92, 93).

#### Role of T Cells

One of the key features of human SS is the abnormal activation and expansion of autoreactive T cells (36, 114, 142), suggesting an important role of T cells in disease pathogenesis. This idea is strongly supported by findings from animal models. For example, T cells are the predominant cell type in the inflammatory cell infiltrates in exocrine glands in all mouse models, consistent with observations in human SS. Furthermore, mouse models of SS provide direct evidence for an essential role of T cells in disease pathogenesis. First, the transfer of T cells from diseased mice in multiple models induced SS-like disease in recipient immunodeficient mice (37, 71, 81, 126), suggesting that T cells are sufficient to induce disease. Second, deficiencies in T cells or T cell–associated cytokines, e.g., IL-17, prevented mice from developing SS-like disease (85, 96, 114), demonstrating that T cells are indispensable for disease development. Third, mice with a T cell–specific *PI3K* deficiency spontaneously developed SS-like disease, showing that abnormalities in T cells mediate disease manifestation. Finally, several mouse models have shown that autoreactive T cells against M3R (126) and 120 kD α-fodrin (81) are potentially pathogenic.

In addition to autoreactive T cells, Tregs have been shown to contribute to disease development in mouse models, which is best exemplified by the NFS/sld, IQI/Jic, and *Ar* KO mice (23, 37, 58, 69). In these genetic models, mice that were thymectomized (Tx) 3 days after birth to eliminate Tregs developed severe disease symptoms, suggesting a protective role of Tregs.

#### Role of B Cells and Autoantibodies

Findings from animal models also demonstrated an essential role of B cells in the development of SS-like disease. On the one hand, genetic modifications directly affect B cell function; for example, mice with *Act1* deficiency (95) and BAFF overexpression (103) spontaneously develop SS-like symptoms, demonstrating that B cell dysregulation could lead to SS-like disease. On the other hand, B cell deficiency mediated either by genetic modification (54) or by anti-CD20 antibodies (26, 130) inhibits disease development in multiple models, suggesting an indispensable role of B cells. Because B cells are involved in both antibody production and antigen presentation, an essential role of B cells in mouse models of SS indicates but does not necessarily confirm that autoantibodies are pathogenic. In 1998, Robinson et al. reported that the transfer of IgG isolated from NOD mice but not normal mouse IgG could decrease saliva production (54), demonstrating for the first time the pathogenic role of autoantibodies in animal models. Notably, B cell–deficient NOD mice, NOD.Igμ^null^ mice, develop exocrine gland lesions similar to those observed in NOD mice but show normal secretory function (54). These findings suggest that B cells are not required for the formation of inflammatory cell infiltrates, but play an indispensable role in the impairment of secretory function via autoantibody production.

### Role of Dysfunctional Homeostasis of Glandular Epithelial Cells

As a consequence of disease manifestation, exocrine gland dysfunction is a key feature of SS with more than 90% of patients exhibiting impaired secretory function of the salivary and/or lacrimal glands (143). Interestingly, evidence from mouse models of SS suggests that dysregulation of exocrine gland homeostasis also contributes to the development of the disease.

#### Glandular Epithelial Cell Apoptosis

In human SS, both the salivary and lacrimal glands exhibit characteristic apoptosis of glandular epithelial cells (144). This glandular epithelial cell apoptosis has been observed in many mouse models of SS, for example, in NOD-derived strains (145–147), *Ar* KO mice (37, 77), *RbAp48-tg* mice (80), NFS/*sld* mice (60, 61), STAT3 KO mice (109), and *TSP-1* KO mice (92). Notably, in these mouse models, epithelial cell apoptosis is an event preceding autoimmune responses, suggesting that it might be the causal factor of autoimmunity. This hypothesis is well-supported by findings in *RbAp48-tg* mice as mice overexpressing the gene in a salivary gland–specific manner showed tissue-specific epithelial cell apoptosis in the exocrine glands (80). Subsequent to epithelial apoptosis, *RbAp48-tg* mice show autoreactive T cells and autoantibodies, which lead to further histological lesions and exocrine gland dysfunction (80). Therefore, the findings in *RbAp48-tg* mice clearly demonstrate that the apoptosis of glandular epithelial cells can trigger autoimmune responses in SS.

The mechanism underlying epithelial cell apoptosis–triggered autoimmunity has also been partially explored in mouse models of SS. In multiple mouse models characterized by epithelial cell apoptosis, autoantibodies against the proteolytic fragments of 120 kD α-fodrin are observed (37, 60, 61, 77, 80), suggesting that autoimmunity against α-fodrin is mediated by epithelial apoptosis. Moreover, the increase in apoptotic epithelial cells is associated with increased proteolysis of 120 kD alpha-fodrin in the salivary glands of *Ar* KO mice and NFS/*sld* mice (61, 77). In addition, increased α-fodrin proteolysis led to enhanced T cell responses against α-fodrin, and passive transfer of α-fodrin–reactive T cells could induce inflammatory lesions in the exocrine glands of recipient mice (37). Therefore, these findings suggest that the axis of glandular epithelial apoptosis/α-fodrin proteolysis/autoimmunity against α-fodrin plays an essential role in the development of SS-like disease.

#### Ectopic Expression of MHC II Molecules

Ectopic expression of MHC II molecules on glandular epithelial cells is another histological characteristic of SS (8). In 1997, Saegusa et al. reported that IQI/Jic mice show expression of MHC II antigen on ductal epithelial cells in the foci (22). Abnormal expression of MHC II molecules on glandular epithelial cells was also observed in *RbAp48-tg* mice (81). Because both IQI/Jic and *RbAp48-tg* mice are genetic models of SS, these findings suggest that this ectopic expression is mediated by genetic factors. Regarding the mechanism underlying ectopic expression, IFN-γ produced by salivary gland epithelial cells in *RbAp48-tg* mice (81) plays an essential role in this process because it can induce high expression of interferon regulatory factor 1 (IRF-1) and class II major histocompatibility complex transactivator (CIITA), which are primary regulators of MHC II molecules (39, 148, 149).

In addition to that in genetic mouse models, ectopic expression of MHC II molecules on glandular epithelial cells has been observed in two induced mouse models: the M3R immunization–induced model (126) and the Ro60_316-335 peptide–induced mouse model (132). Interestingly, in the Ro60_316-335 peptide–induced model, ectopic expression of MHC II molecules is caused by the adjuvant and is an early pre-symptomatic event during the development of disease (132), suggesting that (i) ectopic expression of MHC II molecules can also be caused by environmental factors and (ii) ectopic expression of MHC II molecules might be involved in the development of disease.

Due to the essential role of MHC II molecules in antigen presentation, glandular epithelial cells ectopically expressing MHC II molecules might act as APCs and, thus, contribute to the development of SS-like disease. This idea is supported by a previous finding that salivary gland epithelial cells (SGECs) obtained from patients with SS express both MHC II molecules and co-stimulatory cytokines and can mediate the initiation, development, and maintenance of the inflammatory response as nonprofessional APCs *in vitro* (81).

#### Abnormal Proliferation of Glandular Epithelial Cells

In addition to apoptosis and ectopic expression of MHC II molecules, abnormal proliferation of glandular epithelial cells might also promote autoimmunity (2). In the HTLV-1 tax Tg model, young mice showed ductal epithelial cell proliferation in the exocrine glands. As the mice aged, they showed a distorted structure and severe lymphocytic infiltration into the exocrine glands (32). These data indicate that the proliferation and functional perturbation of ductal epithelial cells in the exocrine glands might be the primary event mediating the subsequent autoimmunity and lymphocyte infiltration in SS. The mechanism underlying this phenomenon needs to be further elucidated.

## Conclusion

The short life span, high fertility, genetic similarity to humans, and the fact that the mouse genome can be readily manipulated make the mouse the most common model organism for human diseases, including autoimmune disorders (150, 151).

The existing mouse models have two major limitations. On the one hand, patients with SS are characterized by a broad clinical spectrum, including glandular and extraglandular manifestations as well as the development of B cell malignancy. However, no single mouse model has captured all immunological and clinical aspects of human SS. On the other hand, patients with SS can be categorized as those with mild disease, e.g., patients with only dry eye and/or dry mouth, or those with severe systemic disease, e.g., patients with severe interstitial lung disease. However, most mouse models for SS only develop a mild disease, and there is a lack of mouse models that show symptoms similar to pSS with systemic diseases with the exception of some mouse models for 2ndSS, such as NOD mice and BAFF-*tg* mice, which develop multiple autoimmune disorders. The discrepancies between human disease and mouse models may be due to several reasons. First, species differences between mice and humans in the immune systems and affected tissues might lead to different disease manifestations (151). Second, all mice used for modeling human SS are inbred strains, which cannot mimic the genetic heterogeneity of human patients. Finally, human SS is a complex disease resulting from the interaction of multiple genetic and environmental factors, and most mouse models are either caused by a single genetic variation or induced by immunization with an antigen or viral infection. Thus, an ideal mouse model that mimics the full profile of human SS is still missing. For better modeling of human SS, novel and more representative disease models need to be developed in the future.

Nevertheless, the existing mouse models for SS are important for the investigation of the significance of certain pathways and etiopathogenic factors in human SS. For example, mouse models have already uncovered some potential etiologic factors for SS, including viral infection, estrogen deficiency, and apoptosis of exocrine glandular epithelial cells. Moreover, mouse models have demonstrated that pathogenic autoimmunity in SS can result from dysregulated T cell development or activation, uncontrolled activation of B cells and abnormal function of APCs. In addition, induced mouse models suggest that autoimmunity to salivary gland proteins, M3R, Ro60, and CAII might play a role in the development of SS. With regard to pathogenesis, Th17 cells; B cells; and some inflammatory cytokines, such as IL-8, IL-17, and IFN-γ, have been suggested to play an important role in disease manifestations (152).

In conclusion, SS is a highly complex and heterogeneous autoimmune disease with multifactorial etiology. Often, human SS coexists with other autoimmune diseases, such as RA or SLE, which are additional obstacles to exploring the pathogenesis of the disease. Mouse models are powerful tools for exploring the etiology and pathogenesis of human SS. In the past two decades, significant progress has been achieved in the field of mouse models of SS, which has helped elucidate the nature of the disease. Research has shown that both abnormalities in the immune system and dysregulated homeostasis in the exocrine glands are involved in the development of SS. Additional and more representative mouse models will provide a better research tool for exploration of the nature of SS.

## Author Contributions

All authors listed have made a substantial, direct and intellectual contribution to the work, and approved it for publication.

## Conflict of Interest

The authors declare that the research was conducted in the absence of any commercial or financial relationships that could be construed as a potential conflict of interest.
